# Extended duration of direct hemoperfusion with polymyxin B-immobilized fiber column improves hemodynamics in patients with septic shock

**DOI:** 10.1186/cc9541

**Published:** 2011-03-11

**Authors:** C Yamashita, Y Takasaki

**Affiliations:** 1 Uwajima Social Insurance Hospital, Uwajima, Japan; 2 Uwajima Municipal Hospital, Uwajima, Japan

## Introduction

Endotoxin adsorption therapy by direct hemoperfusion with a polymyxin B-immobilized fiber column (PMX-DHP) has been widely used in patients with septic shock in Japan. Many Japanese doctors use each PMX cartridge only for 2 hours; however, the mechanisms and optimal duration of PMX treatment remain unclear. We have performed PMX-DHP for longer than 2 hours to confirm that an extended duration of PMX-DHP for patients with septic shock would give significant improvements of hemodynamics.

## Methods

We performed an extended PMX-DHP on 13 patients whose hemodynamics did not achieved the target of mean arterial pressure (MAP) >65 mmHg and inotropic score <5.0 at the time point of 2 hours after PMX-DHP. Hemodynamic parameters such as MAP, heart rate and the dose of vasoactive agents were assessed before treatment, 2 hours after the start of PMX-DHP, immediately and 24 hours after completion of PMX-DHP. The following were also recorded during the study: microbiological data, the APACHE II score, the Sequential Organ Failure Assessment (SOFA) score and 28-day mortality.

## Results

APACHE II and SOFA scores were 26.0 ± 9.0 and 10.4 ± 3.0, respectively. The 28-day mortality rate was 15.4%. The average duration of PMX-DHP was 14.9 ± 7.5 hours. PMX-DHP was well tolerated and showed no side effect over extended duration in treatment. MAP was increased: 64.2 ± 8.8 mmHg (baseline), 79.7 ± 10.5 mmHg (2 hours after the start of PMX-DHP), 88.4 ± 13.8 mmHg (immediately after completion) and 89.8 ± 12.8 mmHg (24 hours after completion). The inotropic score was also decreased: 16.4 ± 9.2 (baseline), 13.5 ± 7.2 (2 hours after the start of PMX-DHP), 5.7 ± 6.8 (immediately after completion) and 2.8 ± 3.6 (24 hours after completion). These improvements for 2 hours were statistically significant (*P *< 0.01).

## Conclusions

The hemodynamics kept improving during extended duration of DHP with one PMX cartridge. And we could use these cartridges safely. Thus we suggest that an extended duration of PMX treatment affords beneficial effects and may contribute to improve the mortality of patients with septic shock.

